# Evidence-Based Supplementation Strategies for Wrestlers: A Systematic Review

**DOI:** 10.1007/s13668-025-00672-x

**Published:** 2025-06-25

**Authors:** Michelle Coutiño Díaz, Arnold Prieto Martínez, Reza Zare, Ali Ali Redha, Scott C. Forbes

**Affiliations:** 1 School of Medicine and Health Sciences, Tecnológico de Monterrey Campus Guadalajara, Jalisco, Mexico; 2 SRH Campus Hamburg, SRH University of Applied Sciences Heidelberg, 20095 Hamburg, Germany; 3 The Department of Public Health and Sport Sciences, Faculty of Health and Life Sciences, University of Exeter Medical School, University of Exeter, Exeter, EX1 2LU UK; 4 Centre for Nutrition and Food Sciences, Queensland Alliance for Agriculture and Food Innovation (QAAFI), The University of Queensland, Brisbane, QLD 4072 Australia; 5 Department of Physical Education Studies, Faculty of Education, Brandon University, Brandon, MB Canada

**Keywords:** Wrestling, Sports nutrition, Exercise performance, Ergogenic aids

## Abstract

**Background:**

Wrestling is a popular combat sport that requires muscular strength, power, agility, and endurance. Weight classes have motivated wrestlers to compete at a lower weight to optimise power-to-weight ratio and performance. To achieve these characteristics, athletes may use dietary supplements, however, their efficacy in wrestlers has not been systematically evaluated.

**Objective:**

The purpose was to systematically review the literature to determine the efficacy of dietary supplements to improve body composition, physiological status, and performance in wrestlers.

**Methods:**

A systematic search was conducted in PubMed, ProQuest Medline, Web of Science, Cochrane Library, and Scopus on the 21st of January 2024 and updated on the 6th of January 2025. Studies were included if the participants were healthy wrestlers ingesting any type of dietary supplement in comparison to a control. Data associated with intervention type and characteristics, target populations, outcomes, and analysis methods were extracted.

**Results:**

A total of 24 eligible original articles were included that assessed various supplementation strategies on body composition, exercise performance, and metabolic markers in wrestlers. Individual studies revealed significant effects of sodium citrate, creatine monohydrate, spirulina, green tea and oolong tea extracts, and branched-chain amino acids on body mass or composition. β-Hydroxy-β-methylbutyrate (HMB-FA), creatine monohydrate, and iron supplementation improved recovery and may improve exercise performance. Beet-root juice supplementation enhanced muscular strength and balance. BCAA supplementation produced mixed results on muscle damage biomarkers and performance, while sodium citrate, creatine, and spirulina can act as buffering agents. Thyme tea appears to improve antioxidant capacity.

**Conclusions:**

Overall, individual studies show some promise for several dietary supplements to alter body mass and body composition, improve exercise recovery and performance, delay fatigue, and modify serum biomarkers; nevertheless, effect sizes were often small, and results were often mixed.

## Introduction

Wrestling is one of the oldest sports, dating back to ancient Egypt and Greece [1]. Currently, wrestling is one of the most popular sports in the world, having more than 1.9 million individuals engaged in wrestling in the U.S. alone [2]. Since competitors are divided into weight classes, this has motivated wrestlers to compete at a lower weight to optimise power-to-weight ratio, body composition, and performance [3]. However, the methods used to alter body mass (BM) and composition are often non-evidence based such as severely restricting food intake, dehydration, or excessive workouts [4, 5]. Furthermore, unlike other combat sports, wrestlers typically compete much more frequently [6] thereby inducing repeated changes in weight that have been linked to a reduction in anaerobic exercise performance, depleted muscle glycogen, reduction in lean body mass, depression, and fatigue [7]. In addition, rapid weight loss achieved through the reduction of energy and fluid intake, as well as increased exercise, increases fatigue and decreases peak power [8].

Multiple factors such as energy production, hormones, inflammation, and oxidative stress could influence a wrestlers’ physical performance. As a high-intensity sport, wrestlers depend on glycolysis to provide the necessary energy to the muscles [9]. In anaerobic conditions, this leads to muscle acidification through the accumulation of hydrogen ions (H^+^), which is associated with muscle fatigue [10]. The physiological process of acidification and muscle fatigue can be explained by: 1) the competition of H + ions with calcium ions (Ca^2+^) for the troponin binding site that prevents contraction from occurring properly; 2) the inhibition of phosphocreatine resynthesis; 3) the inhibition of key enzymes (e.g., phosphofructokinase) of the glycolytic pathway [11]; 4) and a decreased production of energy in muscle cells due to a reduced proton gradient between the mitochondrial matrix and the cellular cytoplasm [12]. Therefore, maintaining a pH in physiological ranges is critical for sustained muscle contractions to occur [13]. During high-intensity exercise, intramuscular acidity is regulated both intra and extracellularly with bicarbonate being one of the major contributors [14, 15]. Oral supplementation with sodium bicarbonate leads to alkalemia, which creates a greater efflux of H^+^ and lactate out of the active muscles and into the circulation [16]; further metabolic alkalosis results in the acceleration of glycogenesis [17] and may potentially reduce membrane depolarization that could lead to increased performance [18]. As such, supplementation with sodium bicarbonate could improve performance due to buffering with H^+^ and the efflux of lactate from the muscle [16, 19].

The physiological stress that wrestlers undergo can also affect the endocrine system, which has been described for testosterone and cortisol levels. In summary, testosterone is an anabolic hormone which promotes muscle and bone mass; however, endogenous production plays a small role in muscle adaptation in comparison to the muscle’s androgen receptor content [20]. Cortisol is a catabolic hormone that increases during physiological stress and stimulates glucose production, decreases amino acid uptake by the muscle, and reduces muscle and bone formation [21]. Elevations in total testosterone and cortisol have been observed in competitive wrestling [22–24], with the testosterone response reducing with subsequent tournaments. This is important given that the response of testosterone has been observed to be greater in the winners [22–24].

Wrestling has also been associated with abnormal levels of inflammatory and oxidative stress markers. Studies have reported that interleukin 1 beta (IL-1B), interleukin 6, tumour necrosis factor-alpha (TNF-a), glutathione peroxidase, superoxide dismutase, lipid hydroperoxides, and total glutathione levels, are altered through training in wrestlers [25]. Some dietary supplements have been purported to help athletes with oxidative stress and inflammatory-related damage, enhance performance and improve recovery. For instance, blueberry supplementation might reduce inflammatory and oxidative stress markers, as well as enhance recovery after exhaustive exercise [26], vitamin D supplementation might be useful to increase strength, lower inflammatory markers, and decrease the risk of injury [27], and zinc supplementation might improve appetite, increase BM, reduce fatigue, and increase endurance [28]. Interestingly, some studies have reported that a proper diet might be just as effective (or more) than supplementation with creatine or glutamine in improving exercise performance [29].

Other commonly used supplements include vitamins, minerals, botanicals or herbs, botanical compounds, amino acids and their derivatives, amongst others [30]. Despite safety and efficacy concerns for some, dietary supplements are generally considered to be part of a well-rounded approach to BM management, and using dietary supplements to modify an individual's body composition has become a common strategy when attempting to alter BM or body composition [31, 32]. As such, while supplementation promises great benefits, there is a need to investigate the efficacy in specific populations. Therefore, in this systematic review, we evaluated studies examining the effects of dietary supplements on markers of inflammation, oxidative stress, body composition, exercise performance, and recovery in wrestlers.

## Methodology

This systematic review was pre-registered in the PROSPERO database (ID:CRD42023458266) and was performed according to the recommendations established by the Preferred Reporting Items for Systematic Reviews and Meta-Analysis (PRISMA) [33].

### Literature Search

An extensive search in PubMed, ProQuest Medline, Web of Science, Cochrane Library, and Scopus was conducted on the 21 st of January 2024 and then updated on the 6th of January 2025 to find studies that assessed the effects of supplementation on body composition, markers of oxidative stress or inflammation, endocrine responses, exercise performance, muscle damage, and recovery in wrestlers. The search expression consisted of: (“wrestlers” OR “wrestling”) AND (“supplement” OR “supplementation” OR “oral”) AND (“weight” OR “body” OR “composition” OR “antioxidant” OR “oxidative stress” OR “inflammation” OR “anti-inflammation*” OR “hormone” OR “muscle” OR “strength” OR “recovery” OR “performance” OR “aerobic” OR “anaerobic” OR “power” OR “exhaustion”). Articles relevant to our investigation that were referenced in any of the included studies were also taken into consideration. No limitation was made on the publication date or time length of the studies. Only studies in English were included. The search strategy and inclusion/exclusion criteria based on population, intervention, comparison, outcomes and study design (PICOS) have been summarised in Table 1.

**Table 1:** Search strategy and inclusion/exclusion criteria based on population, intervention, comparison, outcomes and study design (PICOS)

Databases	Search Terms	PICOS	Inclusion criteria	Exclusion criteria
PubMed, ProQuest Medline, Web of Science, Cochrane, Scopus	(“wrestlers” OR “wrestling”) AND (“supplement” OR “supplementation” OR “oral”) AND (“weight” OR “body” OR “composition” OR “antioxidant” OR “oxidative stress” OR “inflammation” OR “anti-inflammat*” OR “hormone” OR “muscle” OR “strength” OR “recovery” OR “performance” OR “aerobic” OR “anaerobic” OR “power” OR “exhaustion”)	Population	Wrestlers between 18 to 45 years old	Unhealthy individuals; individuals below or above the established age range; other types of athletes or non-sport practitioners
		Intervention	Any dietary supplement	Multiple ingredients used as a single intervention; non-dietary supplementation
		Comparison	Supplementation vs. no supplementation/placebo	
		Outcome	Changes in markers of physiological status, body composition, muscle damage, exercise performance and/or recovery	Alterations in any of the markers before the intervention
		Study design	Randomized, non-randomized, controlled, crossover, and quasi-experimental studies	Meta-analysis, systematic review, cross-sectional, case–control, case reports, animal, and in vitro research studies

### Study Selection

Included studies were randomized and non-randomized controlled trials in humans that had a control group as a comparator to assess the beneficial effects of dietary supplements on markers of physiological status, exercise performance, and recovery. The participants of all the studies were wrestlers. The excluded studies were observational, animal, and in vitro studies. Two reviewers independently assessed the titles and abstracts against the inclusion and exclusion criteria. The eligible full-text articles were retrieved. The full-text screening was completed independently by the two reviewers. Any disagreements were resolved by establishing a consensus.

### Data Extraction

The following data was retrieved from each study: type of intervention, target population characteristics, outcomes, and analysis of the outcomes. All data was summarised and described as qualitative and quantitative variables. A narrative synthesis was performed for the demographic characteristics of the participants such as age, sex, health status, and exercise performance activity, the characteristics of the interventions such as dose, frequency, and intervention time of the supplementation, as well as the characteristics of the placebo and the assessment tools used to determine the antioxidant, anti-inflammatory, and muscle damage markers, as well as recovery time, quantitative and qualitative exercise performance assessments.

### Risk of Bias Assessment

The scientific quality of the studies was assessed independently by two reviewers using the Risk of Bias 2 tool (RoB2) for randomised and crossover trials [34], and the ROBINS-I tool for non-randomised trials [35]. The assessment of randomised trials was based on the following domains: randomisation process, assignment and adherence to intervention, missing data, measurement of outcome, and selection of the reported results. For crossover studies, the risk of bias arising from period and carryover effects was also considered. For non-randomised studies, the assessment also evaluated the bias due to confounding in addition to the domains stated for randomised studies. The studies were then categorised as having a low, some concerns, or high risk of bias. If assessment outcomes were conflicting, reviewers discussed and came to a consensus. Visualisation of the risk of bias assessments was performed using the robvis online tool [36].

## Results

### Study Selection

**Fig. 1:**
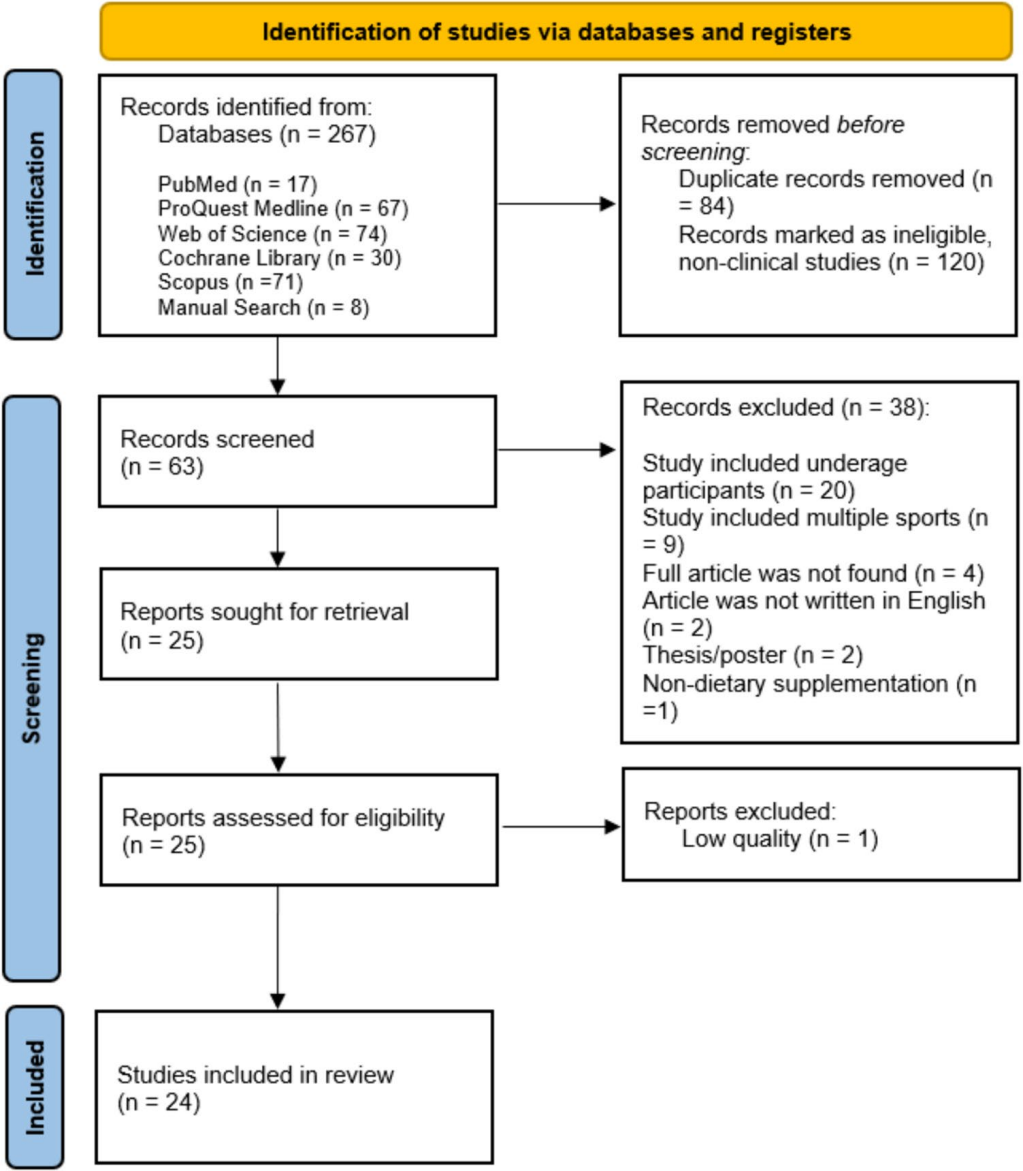
Preferred reporting items for systematic reviews and meta-analyses (PRISMA) flow diagram

The review identified 267 records by searching the five databases. After removing duplicates (n = 84), 183 articles remained, from which 120 non-clinical trial articles were identified and removed before the screening. A total of 63 articles were then screened by title, abstract, and keywords by the reviewers independently. A total of 39 articles were excluded for different reasons: study included underage participants (n = 20), study included multiple sports (n = 9), the complete article was not found (n = 4), the article was not in English (n = 2), the study was a thesis/poster (n = 2), the study used non-diet supplementation (n = 1), or study was of low quality (n = 1). A total of 24 articles were assessed for eligibility. The details of the study selection process are shown in Fig. 1.

### Characteristics of the Included Studies

Among the included studies, one study was a non-randomised, double-blind, placebo-controlled trial [37]; five studies were randomised, double-blind, placebo-controlled trials [38–42]; eight studies were randomised, single-blind, placebo-controlled trials [43–48]; and ten studies were placebo controlled, crossover trials [49–56]. The total supplementation duration ranged from an acute dose 30 min before the test to 14 weeks. Supplementation was given as a single dose or up to four times a day [29, 37–43, 45–53, 56–58]. Nine articles reviewed the effects on body composition [37, 38, 40, 42, 43, 47, 50, 56, 57]; 17 studies evaluated the effects on exercise performance or muscle damage [29, 37–39, 41, 43, 45, 48–52, 54–58], 11 studies analysed the effects on hormonal and metabolic markers [37, 38, 40, 42, 46, 50, 52–55, 57], and six articles reported other outcomes [40, 46–48, 55, 56]. The details of supplementation used in each study are described in Table 2.

**Table 2:** Summary of clinical-based trials evaluating the effect of dietary supplementation on physiological status and/or performance-related outcomes of wrestlers

Supplement	Study	Study design	Participants	Supplemented group	Placebo/control group	Duration	Measured outcomes	Training Load	Key Findings
Sodium citrate	Timpmann, S., et al., 2012	Non-randomised double-blind placebo controlled parallel trial	n = 16 Experience: 5–13 years of training Sex: N/S Age: 18–26 years	n = 8 Controlled diet with a single dose of 600 mg/kg	n = 8 Controlled diet with a single dose of wheat flour	Acute dose 16 h before testing	BM USG MP PP FI Blood pH Blood HCO_3_ BE Blood lactate	UBISP test	**BC:** The average gain in BM was significantly greater in the CIT group *(p* = *0.008)* with a significant interaction effect between test and treatment *(p* = *0.006).* No difference between groups in mean USG **MDEP:** No significant difference in MP, PP, or FI between groups **HMM:** Significantly higher HCO_3_– concentrations *(p* = *0.018)*, higher pH levels *(p* = *0.001)*, and significantly higher base excess in the CIT group *(p* < *0.0001)*
Chromium picolinate	Walker, S., et al., 1998	Randomised double-blind placebo-controlled parallel trial	n = 20 Experience: NCAA division I Sex: male Age: 18–23 years	n = 7 Gelcap with 200 μg once a day	n = 7 Gelcap with sodium diphosphate n = 6 No supplementation	14 weeks	Insulin Glucose BM LBM FM Fat % Leg power Upper body power endurance Peak AnP Rel AnC Rel VO_2_max	Progressive resistance training program and metabolic conditioning regimen Bruce protocol Wingate 30-s cycle ergometer test Upper body endurance (maximal repetitions of seated low-pulls) Absolute lower body endurance (maximal repetitions of leg press performed) Global muscular power (Olympic power clean) Maximal upper body strength (1RM bench press)	**BC:** No significant differences in BM, LMB, Fat %, or FM between groups **MDEP:** No significant differences in leg power, upper body power, endurance, Peak AnP, Rel AnC, and Rel VO_2_max between groups **HMM:** No significant differences in fasting serum glucose levels or insulin concentrations between groups
Creatine monohydrate	Koçak, S., et al., 2003	Randomised placebo-controlled parallel trial	n = 20 Experience: Turkish National Team Sex: male Age: 22–27 years	n = 10 5 g four times a day dissolved in 250 ml of water 1 h prior to eating	n = 10 5 g of milk powder four times a day dissolved in 250 ml of water 1 h prior to eating	5 days	BM AP PP	Wingate 30 s anaerobic test	**BC:** Significantly higher weight gain in CM group *(p* < *0.01)* **MDEP:** Significant difference between pre-and post-test scores of AP *(p* < *0.01)*, PP *(p* < *0.01)*
Creatine monohydrate	Mohamed, E., and Tammam, A., 2020	Randomised, double-blind placebo controlled trial	n = 16 Experience: Shooting wrestling club Sex: N/S Age: 21–23 years	n = 8 1 st week: 20 g of CM dissolved in 250 ml of mil juice for four days Week 2–8: 5 g of CM dissolved in 250 ml of mil juice for 33 days (245 g in 8 weeks)	n = 8 250 ml of mil juice	8 weeks	CPK Muscular endurance Power Agility	Back-throw dummy test Bridge skill test Performance of the skill of the bridge test	**MDEP**: Statistically significant effects of the time (i.e. pre- to post-training) for all measured variables (*p* ≤ 0.05) in the CM group. Statistically significant differences for CPK (*p* = 0.000), muscular endurance (*p* = 0.002), power (*p* = 0.003), and agility (*p* = 0.036) in the CM group at the POST evaluation
Creatine monohydrate + glucose	Ööpik, V., et al., 2002	Randomiseddouble-blind placebo controlled crossover trial	n = 5 Experience: 7 years average Sex: male Age: 19–21 years	n = 5 80 g of glucose + 7.5 g of CM four times a day dissolved in 300–350 ml of natural fruit juice	n = 5 320 g of glucose four times a day dissolved in 300–350 ml of natural fruit juice	Acute dose immediately after the first test (17 h before the final test) The next two doses, three and six hours after the first test, respectively The last dose, at least two hours before the final test	BM Submaximal work Wtot Wmax	Isokinetic performance of the knee extensors for 5 min	**BC:** No effect of treatment on the extent of BM regained during 17 h recovery **MDEP:** Significant increase in Wtot from test 2 to test 3 in CM + GLC trial. A 13.8%—44.5% increase in Wmax in several time points in glc + cr trial *(p* = *0.02)* **HMM:** No significant difference in ammonia, lactate, glucose, or urea levels between groups
Carbohydrates/ Creatine monohydrate/Glutamine	Abbasalipour, M., et al., 2012	Randomised, single blind parallel group controlled trial	n = 28 Experience: elite wrestlers Sex: male Age: 18 to 25 years	n = 7 Carbohydrate solution made of 5% honey n = 7 0.3 g/kg of CM per day n = 7 Glutamine supplement 0.3 g/kg/day	n = 7 Control group	15 days	Grip strength Agility	Bicycle ergometer till the point of complete exhaustion 9 × 4 agility test Hand grip	**MDEP**: Significant increase in grip strength and agility performance in CM group (*p* < 0.05)
Creatine Monohydrate	Zahabi, G., et al., 2024	Randomised, single blind parallel group controlled trial	n = 18 Experience: International/professional wrestlers Sex: female Age: 18 to 19 years	n = 6 Loading phase (5 days): 5 g of CM four times per day Maintenance phase (20 days): 5 g of CM 30 min before and immediately after training	n = 6 training without supplementation n = 6 no training, no supplementation	25 days	BM BMI BF% FFM VO_2_max RHR 1RM Agility Muscular power	Resistance training Chest and leg press Modified Bruce protocol Chest and leg press IAT VJT	**BC:** Significant increase in BM and BMI in CM group **MDEP**: Significant increase in VO_2_max, 1RM, agility, and muscular power in CM group
Citrulline Malate	Jafari, R., et al., 2024	Randomised, crossover trial	n = 12 Experience: Varsity Junior team 5 + years Sex: N/S Age: 18 to 21 years	n = 12 8 g of citrulline malate	n = 12 N/S	Acute dose one hour before testing	Hypoxanthine HGPRT Grip strength BAT test BLC strength test RPE	JSWPT protocol Hand grip Burpees Isometric force with a dynamometer	**HMM:** No significant differences in hypoxanthine or HGPRT levels **MDEP:** No significant differences in grip strength, BAT test, or BLC strength test
Arginine	Ulas, H., et al., 2012	Randomisedplacebo-controlled crossover trial	n = 10 Experience: National and international level Sex: male Age: 20–28 years	n = 10 1.5gr/10 kg capsules after a 12 h fasting once a day	n = 10 Capsules with starch	Acute dose 60 min before the test and after an overnight fast	Amino acid levels	Cycle ergometer Warm up at 60 rpm without any load for 3 min, followed by 90 watts load increasing 30 watts every 3 min till the exhaustion	**HMM:** Significantly higher pre- and post-exercise arginine, ornithine and citrulline concentrations in arginine trial (p < 0.05) Higher post-exercise TAA, BCAA, glutamine, tyrosine, methionine, phenylalanine, isoleucine and leucine concentrations in arginine trial (p < 0.05)
Arginine	Yavuz, H. U., et al. 2014	Randomised crossover trial	n = 9 Experience: national and international level wrestlers Sex: male Age: 24.7 ± 3.8 years	n = 9 Single dose of 1.5 g/10 kg body weight arginine capsules	n = 9 Equal number of capsules containing starch	Two weeks One week wash-out period	Lactate HR Time to exhaustion Maximun oxygen consumption	Incremental bicycle ergometer test to exhaustion	**HMM:** No significant difference in mean lactate levels **MDEP:** No significant difference in maximum oxygen consumption or in maximum heart rate Time to exhaustion was longer with arginine supplementation compared to placebo (*p* < *0.05*)
Arginine	Zembron, A., et al., 2020	Randomised, double-blind, parallel group, placebo-controlled trial	n = 32 Experience: Members of the Polish national team Sex: male Age: 20 to 29 years	n = 7 Arginine 2 × 6 g per day n = 9 Hypoxia and arginine 2 × 6 g per day n = 6 Hypoxia	n = 10 Placebo 2 × 6 g per day	12 days	BM FFM FM CK NO H2O2 CRP HGF IGF-1 PDGF VEGF BDNF TC HDL LDL TG Hb RBC RET HTC MCV MCH MCHC RDW	14-day training camp	**BC:** No significant difference between groups **HMM:** No significant differences between groups **OO:** NO levels significantly increased in arginine group *(p* < *0.05)*
Carbohydrate, BCAAs and arginine	Jang, T. R., et al., 2022	Randomised,double-blind, cross-over trial	n = 9 Experience: At least 4 years and experience in national or international competitions Sex: male Age: 19.2 ± 0.4 years	n = 9 1.2 g/kg glucose (CH trial) 1 g/kg glucose + 0.1 g/kg Arg + 0.1 g/kg BCAA (leucine: isoleucine: valine = 2:1:1, CH + AA trial)	n = 9 600 ml lemon flavored water	One day Wash-out period of at least 2 weeks	PP MP Glucose Insulin Glycerol NEFA Lactate	3 wrestling matches	**MDEP:** No significant differences between groups **HMM:** Significantly higher glucose and insulin levels in CH + AA trial and lower glycerol and non-esterified fatty acid concentrations
BCAA	Armisaran, R., et al., 2014	Randomised placebo-controlled semi-experimental trial	n = 29 Experience: trained Mahabad City wrestlers Sex: N/S Age: 22 years	Low Dose n = 10 68 mg/kg × 3 times/day before meals for six days before the test and two days after 210 mg/kg 30 min before and after the test High Dose n = 10 68 mg/kg × 3 times/day before meals for six days 450 mg/kg 30 min before and after the test	n = 9 Dextrin 68 mg/kg three times a day before meals for six days before the test and two days after 210 mg/kg 30 min before and after the test	8 days Acute dose 30 min before and after the test	CK CKMB LDH	80% 1RM leg presses, chest presses, lat pull downs, leg extensions, arm curls, leg curls, and abdominal crunches	**MDEP:** No significant difference in CK, CKMB, or LDH levels between groups
Caloric restriction/BCAA	Mourier, A., et al., 1996	Randomised,single-blind, parallel-group, group controlled trial	n = 31 Experience: wrestlers of the French National Institute of Sports Sex: male Age: N/S	n = 7 Hypocaloric high-protein n = 6 Hypocaloric high-branched-chain amino acid n = 6 Hypocaloric low-protein	n = 6 Normocaloric control n = 6 Hypocaloric control	19 days	BM BMI BF SAT VAT AT MT VO2max MVC Glycerol T3	Treadmill Right knee isometric extensions Arm ergometer Wingate Anaerobic Capacity Test	**BC:** Significantly higher BM loss on hBCAA, with a significant loss on SAT (*p* < *0.05*). Significant effect on time and diet on thigh muscle adipose tissue with a greater loss in hBCAA group (*p* < *0.05*) **MDEP:** No significant differences in VOmax, peak power, or endurance time of the extensor muscles **HMM:** No significant differences in glucose, lactate, or insulin plasma levels
Beetroot Juice	Tatlici, A., et al., July 2021	Randomised double-blind crossover trial	n = 8 Experience: trained wrestlers Sex: male Age: 19–24	n = 8 140 ml of BRJ	n = 8 140 ml of cherry juice with lemon juice	Acute dose 150 min before the test	OSI APSI MLSI	Dynamic and static balance in a biodex balance system Maximal contraction knee extension and flexion	**MDEP:** At rest- static MLSI, dynamic OSI, and dynamic APSI significantly improved in BRJ. *(p* = *0.00, 0.03, 0.01,* respectively) At fatigue- static OSI, static APSI, dynamic OSI, dynamic APSI, and dynamic MLSI significantly improved in BRJ. *(p* = *0.00, 0.01, 0.01, 0.02, 0.02,* respectively)
Beetroot Juice	Tatlici, A., June 2021	Randomised double-blind crossover, placebo-controlled trial	n = 8 Experience: trained wrestlers Sex: male Age: 19–24	n = 8 140 ml of BRJ	n = 8 140 ml of cherry juice with lemon juice	Acute dose 150 min before the test	PE PF PIR PER AE AF AIR AER	Extension and flexion strength of the knee Internal and external rotation strength of the shoulder	**MDEP:** No statistically significant difference in ExtP and FlexP. Significant increase in IntP and ExtP in BRJ. (*p* = *0.048 and p* = *0.024*, respectively) Significant increase in ExtAvg, FlexAvg, IntAvg, and ExtAvg in BRJ (*p* = *0.023, 0.027, 0.023, and 0.021,* respectively)
HMB-FA	B. Tartibian, B. Rezaei, 2021	Randomised double-blind placebo-controlled parallel trial	n = 20 Experience: elite wrestlers Sex: N/S Age: 19–26	n = 10 3 g/d of HMB-FA	n = 10 N/S	Single dose	CK LDH PRS	Five simulated wrestling protocols	**MDEP:** Significantly lower levels of LDH after the first, third, and fifth tests. (*p* < *0.05*) Significantly lower level of CK after the fifth test. (*p* < *0.05*)
Spirulina	Bagheri, R., et al., 2021	Randomised, placebo-controlled double-blind trial	n = 40 Experience: wrestling training at least three times a week for at least 3 years before the study Sex: N/S Age: 22 years	n = 20 Designed diet for weight loss Two tablets containing 500 mg of spirulina	n = 20 Designed diet for weight loss Two tablets containing 500 mg of placebo	12 days	BM BFP FM SMM FST MST FST:MST IGF-1 AST ALT	Daily moderate physical activity lasting 20–40 min, which consisted of technical training	**BC:** Significantly lower BFP, SMM, and FM in SP group *(p* < *0.001)* **HMM:** Significantly lower MST, AST, and ALT concentrations in SP group *(p* = *0.005)*. Significantly lower FST and IGF-1 concentrations in PL group (*p* < *0.05*)
Spatone®	Sung, Y., et al., 2018	Randomised, single-blind, crossover, group controlled trial	n = 9 Experience: amateur wrestlers Sex: male Age: N/S	n = 7 Spatone water containing 5 mg of iron in orange juice, two times a day for 1 week of weight-loss period	n = 6 Weight loss of 7% over 7 days with placebo	7 days 4 weeks wash-out period	BM SMM FM BMI VO2max Lactate Calcium Magnesium Iron RBC Hb Hct MCV Platelet WCV MCH MCHC IL-10 TNF-alpha IL-6	Morning session, consisted of running and dashing, on Monday, Wednesday, and Thursday. The interval training and fartlek training were on Tuesday and Friday For the afternoon session, mat training For the evening session, the subjects performed weight training on Tuesday and Thursday Training protocol twice or thrice per day and the training session that consisted 30 h a week of exercise, 5 h each day	**BC:** No significant differences between groups **MDEP:** Significantly higher endurance capacity, VO2max, and lactate accumulation in Spatone group (*p* < *0.05*) **OO:** No significant differences between groups
Thyme tea	Berkan, C., et al., 2013	Randomised, single-blind, parallel-group, placebo controlled trial	n = 18 Experience: wrestler students of Nidge University Physical Education and Sports School Sex: male Age: 18 to 28 years	n = 9 Thyme tea three times/day and a loading dose of 150 cm3	n = 9 Contol group	35 days	MDA TAC RSH	5 wrestling matches	**HMM:** Significant increase in TAC (*p* < *0.05*) **OO:** Significantly lower MDA levels in thyme group (*p* < *0.01*)
Green tea extract/ Oolong tea extract	Bajerska, J., et al., 2010	Randomised, single-blind, parallel group, placebo controlled trial	n = 35 Experience: Sobiesky Poznan wrestling team Sex: male Age: 18 to 24 years	n = 10 Two capsules three times/day of 400 mg, containing 60% green tea extracts n = 10 Two capsules three times/day of 400 mg, containing 40% oolong tea extracts	n = 10 Two capsules three times/day of 400 mg, containing 100% cellulose	6 weeks	BM FM FFM MEB		**BC:** Significantly lower BM in GTE (*p* < *0.05*) and OTE (*p* < *0.01*) groups. Significantly lower FM in OTE group. (*p* < *0.05*) **OO:** No significant differences between groups
Iron	Sung, J. Y., 2021	Randomised, single-blind, crossover trial, placebo-controlled trial	n = 23 Experience: N/S Sex: 13 males and 10 females Age: 21.6 ± 0.8 years for males and 20 ± 1 for females	n = 23 Water containing 5 mg of iron and orange juice two pouches per day	n = 23 Orange juice	7 days 3 week washout period	BM RBC Hb Hct WCW MCH MCHC TIBC Fe Transferrin EPO VO2max Lactate	Training protocol two or three times each day, totaling around 5 h of exercise each day, and 30 h over the week Morning session, running and dashing on Monday, Wednesday and Thursday. Interval and fartlek training on Tuesday and Friday Afternoon session, mat training Evening session, weight training on Tuesday and Thursday	**OO:** Significantly lower Fe, (p < *0.041)*; transferrin,(*p* < *0.004)*; TIBC (*p* < *0.031*) in males Both groups experienced decreases in erythropoietin (males, *p* < *0.021*; females, *p* < *0.027*) **MDEP:** Significant decrease in VO2max (*p* < *0.001*) after weight loss **HMM:** Blood lactate in the intake group decreased after maximal exercise immediately after the test for the male group (*p* < *0.031*) and during resting time and immediately after exercise in the female group (*p* < *0.001* and *p* < *0.050*, respectively)
Whey protein	Shwawy, A., 2013	Randomised, single-blind, parallel group, group controlled trial	n = 18 Experience: trained wrestlers Sex: male Age: N/S	n = 10 Supplement pre-exercise 1.4 g/kg of BM/day n = 8 Post-exercise supplement 1.4 g/kg of BM/day	n = 18 Control group	Twelve weeks	Total protein Albumin Urea Creatinine Leg extension Barbell Bench Press Barbell Front Raise	Squat, chest and arm exercises	**MDEP:** Significant increase in strength on the barbell bench press (*p* < *0.05*) **OO:** Significant higher total protein and albumin levels (*p* < *0.05*)
Caffeine	Negaresh, et al., 2018	Randomised double-blind,crossover trial, placebo-controlled trial	n = 12 Experience: professional male freestyle, wrestling experience if at least 10 years Sex: male Age: 24 ± 3 years	n = 12 High-dose of caffeine 10 mg/kg 5 times a day n = 12 Moderate-dose of caffeine 4 mg/kg 5 times a day n = 12 Repeated dose caffeine 5 × 2 mg/kg 5 times a day n = 12 Selective caffeine consumption 6.16 ± 1.58 mg/kg 5 times a day	n = 12 Placebo	1 day	PWPT time HR Fatigue rating Lactate Urine osmolality USG	Simulayed wrestling tournament PWPT	**MDEP:** Significantly lower PWPT time before the first match with high dose of caffeine (*p* < *0.05*) Significantly lower PWPT time during the third and fourth matches with repeated doses and selective administration (*p* < *0.05)* Significant time effect for hip/back strength and vertical jump height (*p* < *0.05*) Significantly lower fatigue rating before the fourth match with selective and repeated doses (*p* < *0.05*)

### General Findings

**Fig. 2:**
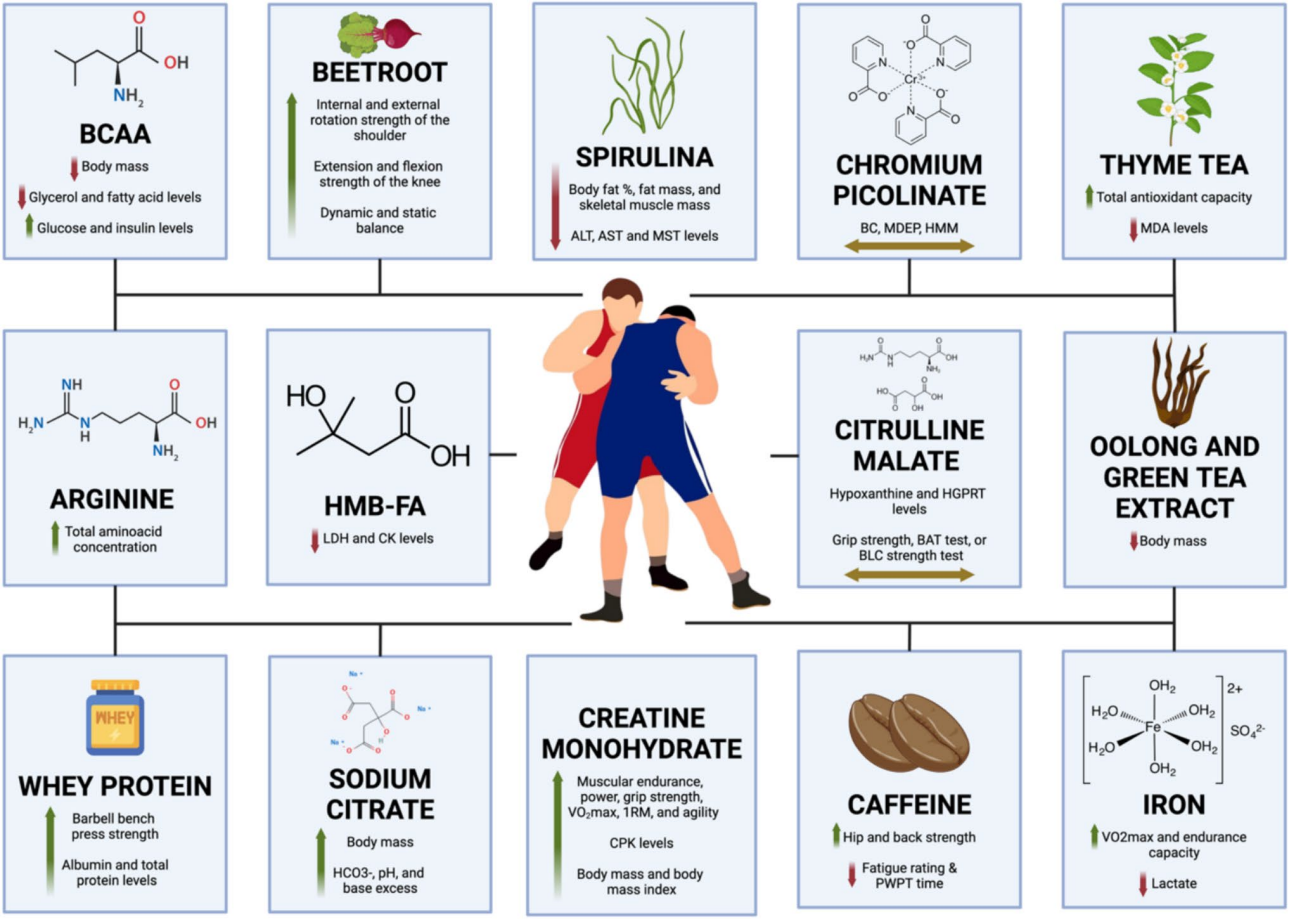
Summary of the effects of different dietary supplements on body composition, exercise performance, and hormonal and metabolic markers

This systematic review included 24 studies (23 randomised and 1 non-randomised trial). The studies included between five to forty wrestlers who were considered healthy and young (18–29 years old). Sixteen studies evaluated men, only one study evaluated women, another one evaluated both men and women, and six did not specify sex. A total of 415 participants were studied, 28 of which were women and 274 were men; the sex of 113 participants was not disclosed. The studies analysed the effects of supplementation after various types of trials such as running, cycling, callisthenics, using exercise machines, wrestling match simulations, and habitual training. The different types of supplementations included sodium citrate, chromium picolinate, creatine monohydrate, arginine, branched-chain amino acids (BCAA), beetroot juice, β-Hydroxy-β-methylbutyrate (HMB-FA), spirulina, Spatone®, carbohydrates, and glutamine. The relevant changes observed with each type of supplementation are discussed throughout Sects. 3.3.1 to 3.3.4 and summarized in Table 2 and Fig. 2 [29, 37–43, 45–54, 56–58].

#### Body Composition

A study evaluating the effects of sodium citrate supplementation reported a higher BM recovery after rapid body mass loss in the experimental group [37]. Neither group achieved the same BM as before the rapid body mass loss, but the deficit was significantly higher in the placebo group. No significant differences between groups were found in underwater weighing, skinfold thickness, body circumference, BM, lean BM, body fat %, or fat mass with Chromium picolinate (CrPic) supplementation [38]. Three studies evaluated the effects of creatine monohydrate (CM) on body composition [59]. Kocak & Karli [43] found a significant increase in the mean BM gained during 5 days of supplementation. Zahabi and colleagues (2024) found similar results in female wrestlers, where 25 days of supplementation significantly increased BM and BMI [59]. Oopik and colleagues (1998) examined acute creatine monohydrate + glucose supplementation but revealed no effect on BM regain during a 17-h rapid body mass loss recovery period [50]. A study investigating the effects of spirulina supplementation found significantly lower body fat %, skeletal muscle mass (SMM), and fat mass in the experimental group [42]. Sung and colleagues (2018) examined Spatone®, which is a natural iron-rich water supplementation, on SMM, body fat, and body mass index (BMI), but found no significant differences between groups [56]. Oolong (OTG) tea extracts were able to significantly influence BM reduction in the oolong group at weeks 4 and 6. The BM reduction was accompanied by a significant decrease in adipose tissue. Additionally, there was a significant difference in the percentage of fat reduction at weeks 2, 4, and 6, as well as in the green tea extract group (GTG) at week 6 [47]. A study found that high doses of caffeine caused a higher urine volume output and dehydration index [49]. Caloric restriction and branched-chain amino acid (BCAA) supplementation were found to have a significant main effect of time on BM reduction; interestingly, the hypocaloric branched-chain amino acid group (hBCAA) was the one with the greatest BM loss, where a significant loss in subcutaneous adipose tissue was found. A significant effect on time and diet was also present on the thigh muscles adipose tissue, where the hBCAA group had a significantly higher loss of adipose tissue than the other hypocaloric groups [57].

#### Muscle Damage and Exercise Performance

Sodium citrate supplementation resulted in no significant differences between groups performing upper body intermittent sprint performance [37]. CrPic supplementation showed no significant differences between groups on peak aerobic power, peak anaerobic power, maximal anaerobic capacity, upper and lower body endurance, upper body power, bench press power, leg press power, or global muscular power [38]. Two studies analysed the effects of BCAA supplementation on muscle damage biomarkers and exercise performance. Amirsasan and colleagues (2011) found no significant difference in creatine kinase (CK), creatine kinase-MB (CKMB), or lactate dehydrogenase (LDH) between groups [45]. Mourier and colleagues (1997) found no significant differences in VO_2_max, peak power output, or endurance time of the extensor muscles with caloric restriction and BCAA supplementation [57]. Jafari and colleagues (2024) found that citrulline malate supplementation had no significant effects on hand grip strength, the back-chest-leg strength test, nor the burpee agility test [60]. Supplementation with β-Hydroxy-β-methylbutyrate (HMB-FA) resulted in significantly lower CK and LDH after simulated wrestling protocols [41]. Four studies evaluated the effects of CM supplementation on exercise performance, all of which reported favourable results. Zahabi and colleagues (2024) reported a significant increase in VO_2_max, one repetition max on chest and leg press, force applied during a vertical jump test, as well as a faster time during the Illinois agility test [59]. Kocak & Karli (2003) reported a significantly higher average power and peak power after supplementation [43]; Oopik and colleagues (1998) reported a significant increase in maximal work (W_max_) and total work (W_tot_); additionally, a strong correlation was established between whole-body creatine retention and the extent of change in W_max_ [50]. Sabry & Tammam (2020) reported significantly better effects of the time for creatine phosphokinase (CPK), muscular endurance, power, and agility [39].

Two studies evaluated beetroot juice supplementation (BRJ). Tatlici and colleagues (2021) found significantly improved balance, resulting in a better at-rest static medial–lateral stability index (MLSI), dynamic overall stability index (OSI), and anterior–posterior stability index (APSI). At fatigue, significant improvements in static OSI, APSI, and dynamic OSI, APSI, and MLSI were also found. The second study reported no statistically significant difference in peak extension and flexion knee strength, however, a significant increase in peak strength of internal and external rotation of the shoulder was found after supplementation. Additionally, all average strength values for extension and flexion of the knee, as well as strength values for internal and external rotation of the shoulder, significantly increased [58].

Two studies analysed the effects of arginine supplementation on muscle damage biomarkers and exercise performance. Yavuz and colleagues (2014) found that supplementation significantly increased time to exhaustion by 5.8% compared to placebo [53]. The second study evaluated the effect of intermittent hypoxic exposure and supplementation with high doses (12 g) of arginine and found no significant differences in CK levels between groups [40]. Iron supplementation was found to either diminish the reduction of VO_2_max after exercise [55] or augment VO_2_max [56]. A study that analysed caffeine intake found that the Pittsburgh Wrestling Performance Test (PWPT) time was lower in the high-dose (10 mg/kg) caffeine group. Additionally, a repeated dose of caffeine as well as selective supplementation, reduced the PWPT times before the third and fourth match. A significant time effect for hip/back strength and vertical jump height was found as well, however, there were no differences in performance between caffeine-intake protocols. Further, the fatigue rating was lower before the fourth match in the selective and repeated dose administration group, while the placebo group reported higher fatigue ratings before the third and final matches [49]. With regards to whey protein supplementation, exercise performance was not improved when taken either immediately after a training session or 40 min prior on leg extensions or barbell front raises; nevertheless, it did increase performance on the barbell bench press [48].

#### Hormonal and Metabolic Markers

Sodium citrate supplementation reported higher pH, HCO3- levels, and base excess in the experimental group [37]. CrPic supplementation reported no significant differences in insulin or glucose concentrations [38]. One study evaluated the effects of CM supplementation on metabolic markers and reported no significant differences in ammonia, lactate, glucose, or urea [50]. Two studies evaluated arginine supplementation on metabolic parameters. The first one reported that the concentrations of glutamine, tyrosine, methionine, phenylalanine, leucine, isoleucine, and total BCAA (branched chain amino acids –valine, leucine, and isoleucine) levels were significantly higher following exercise in the experimental group. Additionally, pre- and post-exercise concentrations of arginine, ornithine, and citrulline were higher in the supplementation group [52]. The second one reported no significant effect on lactate concentrations [53]. Jafari and colleagues (2024) reported that an acute dose of citrulline malate had no effect on hypoxanthine or hypoxanthine–guanine phosphoribosyltransferase (HGPRT) levels [60]. The study evaluating the effects of spirulina supplementation described a significant main effect of time for follistatin (FST) concentrations, a significant group for time effect for myostatin (MST) and FST:MST, as well as significantly lower aspartate aminotransferase (AST) and alanine aminotransferase (ALT) levels. Additionally, the MST concentrations significantly decreased in the supplementation group, the FST:MST ratio was significantly lower in the placebo group, and the FST and insulin-like growth factor 1 (IGF-1) levels significantly decreased in this group as well [42].

Two studies analysed the effects of BCAA supplementation on metabolic markers. Jang and colleagues (2011) investigated the effects of carbohydrate, BCAA, and arginine supplementation and reported significantly higher concentrations of glucose at 30 min and significantly higher insulin concentrations after 30, 60, and 90 min [54]. Mourier et al. (1997) found that the concentrations of glucose, lactate, and insulin in plasma had no significant changes with caloric restriction and BCAA supplementation. Nonetheless, the nitrogen-enriched (N-enriched) diets lowered the concentrations of free fatty acids (FFA) and raised the concentrations of glycerol. Additionally, triiodothyronine (T3) concentrations were significantly lower in the N-enriched diets [57].

Thyme tea supplementation resulted in a significant increase in total antioxidant capacity (TAC) [46]. Iron supplementation significantly lowered lactate levels after 10 min of recovery in the supplement group; interestingly, not only did male participants show a significant decrease in lactate after rapid body mass loss, but they also had significantly higher levels during the resting period and immediately after testing, than women [55]; another trial observed a decrease in lactate accumulation in the early phase after exhaustive exercise but resulted in no significant changes in calcium and magnesium concentrations [56]. Repeated and selective (administration of caffeine based on performance decrement before the wrestling) caffeine supplementation increased lactate levels after the third match. With selective administration, lactate levels were lower before the fourth match, but higher with repeated administration [49].

#### Effects on Other Outcomes

Other outcomes include anti-inflammatory and antioxidant markers, as well as growth factors, protein and albumin levels, and blood parameters. Thyme tea significantly decreased malondialdehyde levels [46]. No significant differences in mean energy balance values were observed after supplementation with GTE or OTE [47]. Iron supplementation resulted in a significant increase in haemoglobin (Hb) and hematocrit (Hct) levels. Interestingly, iron concentrations, transferrin, and total iron bound capacity (TIBC) increased significantly, but only in the male supplement group [55]; further, the proinflammatory cytokines interleukin 10 and 6 (IL-10 and IL-6), and tumour necrosis factor-alpha (TNF-alpha) had no significant changes between groups [56]. Intermittent hypoxic exposure (IHE) and a high dose of arginine significantly increased nitric oxide (NO) and hydrogen peroxide (H_2_O_2_) concentrations, nevertheless, arginine alone was not as effective. A similar pattern was observed with C reactive protein (CRP), multiple haematological markers (i.e., haemoglobin, hematocrit, amongst others), as well as with tissue regeneration mediators such as hepatocyte growth factor (HGF), insulin-like growth factor 1 (IGF-1), platelet-derived growth factor (PDGF), brain-derived neurotrophic factor (BDNF), and vascular endothelial growth factor (VEGF) [40]. A 12-week whey protein supplementation resulted in significantly higher total protein and albumin levels [48].

#### Risk of Bias Assessment

**Fig. 3:**
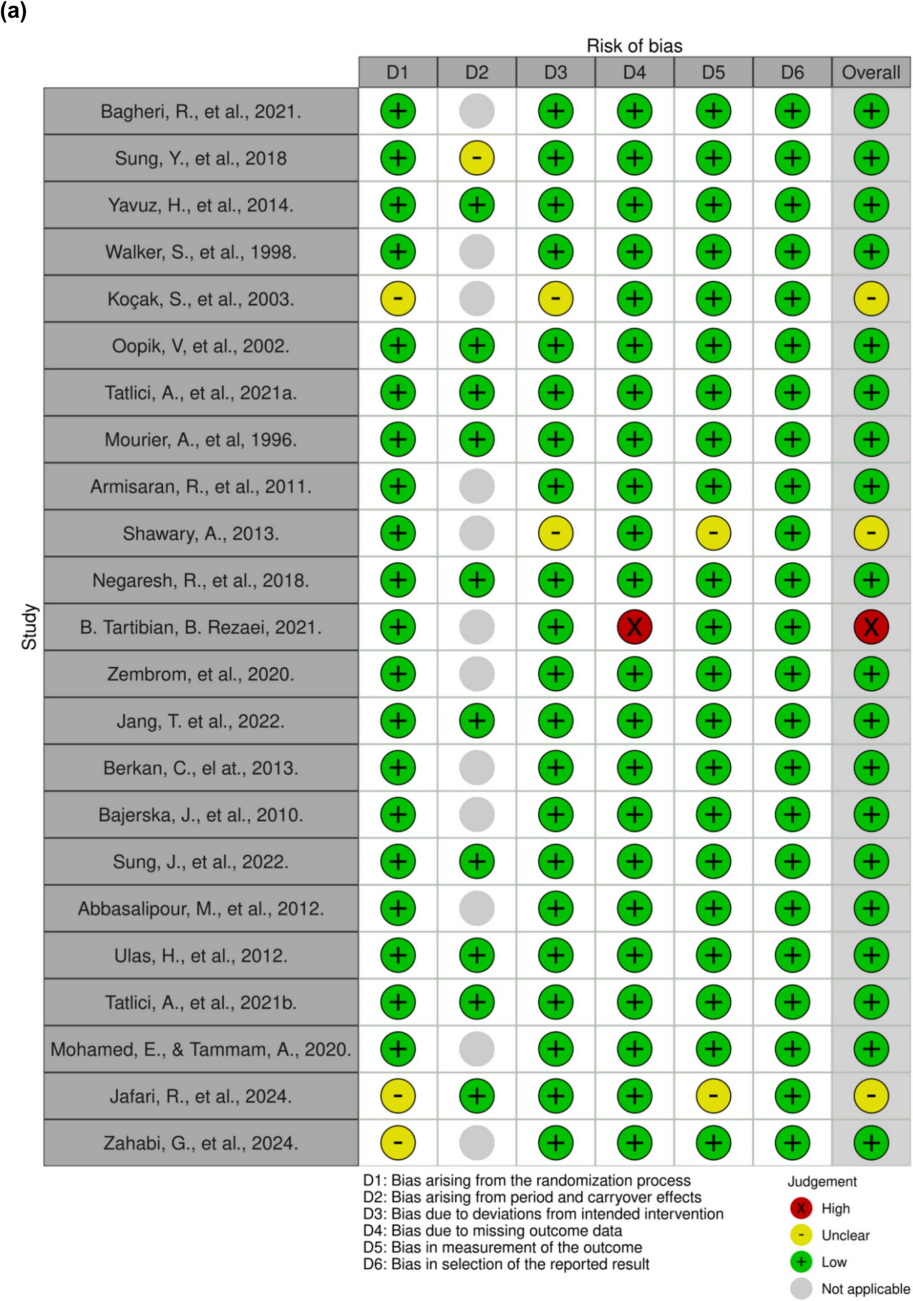

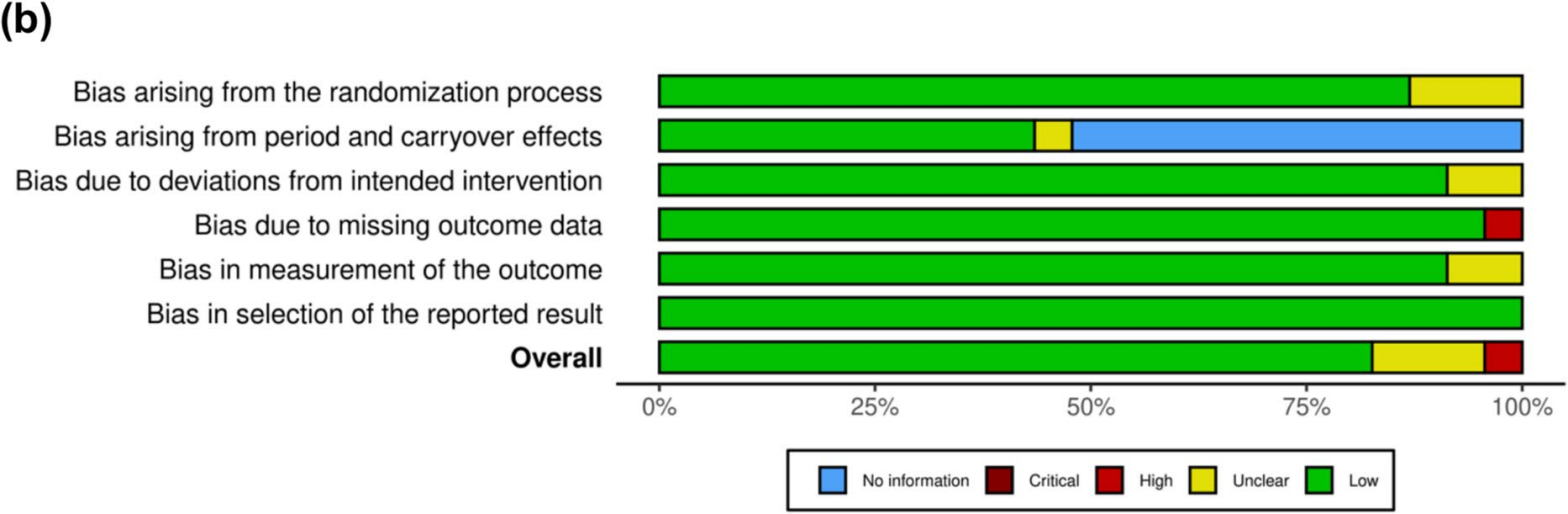
Assessment of bias of the randomized studies according to RoB 2 tool – (**a**) traffic light plot and (**b**) summary plot

**Fig. 4:**
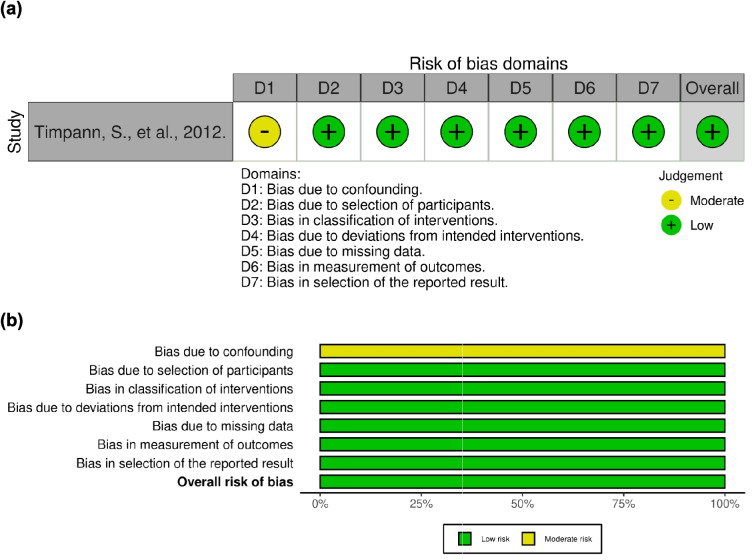
Assessment of bias of the non-randomised studies according to the ROBINS-I tool – (**a**) traffic light plot and (**b**) summary plot

A total of 23 studies were randomised trials and only one was a non-randomised trial [29, 38–43, 45–48, 50–54, 56–58] (Figs. 3 and 4). Out of the 23 studies, one presented a high risk of bias, and three raised some bias concerns [41, 43, 48]. The first one posed a high risk of bias because of missing data. This study evaluated CK, LDH, and perceived recovery status (PRS) but only presented a graph for LDH values over time. No data was presented for the other two variables [41]. The second study raised some bias concerns regarding the randomisation process and deviations from the intended interventions since the participants were not randomly allocated and because there was insufficient information to determine if this deviation arose because of the trial context [43]. The last study raised some bias concerns since it is not clear on how the allocation sequences were concealed, however, baseline imbalances do not suggest a problem. It is also not clear if the outcome assessors were blinded when evaluating results [48]. Other than the lack of randomisation, the non-randomised study was deemed to have a low risk of bias [37].

## Discussion

This systematic review evaluated the effects of dietary supplements on body composition, muscle damage, exercise performance, and hormonal and metabolic markers. Out of multiple ergogenic aids, we found that some of them had significant effects on exercise performance, such as creatine monohydrate [29, 39, 43, 50]; while others had little or no effects, such as BCAA and arginine [45, 54, 57].

### Body Composition

The ingestion of a single 600 mg/kg dose of sodium citrate was useful for regaining BM. Supplementation favours rehydration and restoration of BM through water retention, as well as through a reduced volume of urine excretion within a couple of hours after consumption. This in turn leads to a higher plasma volume (PV), which might be beneficial for the cardiovascular system and exercise performance [61, 62]. In addition, previous reports suggest that lower doses (200–300 mg) are better tolerated and produce less gastrointestinal effects than higher doses (500–600 mg), however this also diminishes the overall effect of supplementation [63, 64].

CrPic supplementation, with a dose of 200 μg/day for 14 days was not able to change body composition in wrestlers. CrPic has been implicated in inducing the effects of insulin, which should in theory promote muscle anabolism via a reduction in muscle protein breakdown [65–67]. Nevertheless, only a few studies have reported desirable effects in body composition with supplementation [68–70]. This might be because of estimation errors associated with the use of tools other than hydrodensitometry to determine body composition or because of the lack of control groups to assess the possibility of a placebo effect. This premise is supported by similar results obtained in other studies where CrPic supplementation coupled with strength training did not result in significant alterations in body fat or lean BM [71, 72].

CM supplementation for five days was able to significantly increase BM. These findings were consistent with other studies where weight gain was reported with dosages ranging from 1.5 to 25 g/d for at least three days in untrained and trained participants [73]. Even high doses (20–25 g/d) for short periods of time have been reported to increase BM between 0.7 to 1.6 kg [74–76]. These effects are due to water retention and/or protein synthesis. However, an acute supplementation of CM + glucose in wrestlers did not accelerate the restoration of BM over 17 h. Potentially, the supplementation duration was too short, however, previous studies have demonstrated that there is a higher accumulation of BM and muscle creatine when combining creatine monohydrate with carbohydrates, nonetheless, it is likely that these effects occur within days, not hours, even when ingested with glucose [77]. These finding might not come as a surprise when other studies analysing a similar dosage for longer periods of time reported only a 0.1 kg/day increase in BM [78, 79]. Another explanation might be that the effect of creatine on BM restoration was masked by the marked (~ 2.4 kg) and fast (17 h) weight increase. In short, the absence of a change in BM does not strictly determine that the creatine supplementation has failed to increase the muscle's creatine content [29].

Spirulina supplementation, with 500 mg/day for 12 days, significantly decreased body fat percentage and fat mass. In theory, these effects are caused by activating an AMP protein kinase signalling pathway and sirtuin 1 in adipose tissue and skeletal muscle. Sirtuin 1 deacetylates nuclear factor-kB (NF-kB), c-Myc, forkhead transcription factor 1 (FKHR), and peroxisome proliferator-activated receptors (PPAR-γ), increasing the levels of adiponectin which in turn decreases fat mass [80–85]. Not only does it raise the expression of PPAR-γ coactivator 1-α and uncoupling protein 2 in skeletal muscle, which results in the up-regulation of adiponectin, but it also promotes mitochondrial biogenesis and fat oxidation [82–84]. Supplementation with oolong tea extracts, with a dose of 2400 mg containing 40% oolong tea for 6 weeks, had a positive effect on BM reduction. While these effects have only been observed in other studies in obese people, these results might be attributable to the effect of catechins present in the tea, which cause an increment in lipid catabolism [86, 87]. Another effect that may contribute is the inhibition of gastric and pancreatic lipases, the modulation of appetite, the stimulation of thermogenesis by the inhibition of catechol-O-methyl-transferase, and the suppression of fatty acid synthesis [88].

Caloric restriction and branched-chain amino acid (BCAA) supplementation were found to have a positive effect on BM reduction, with a high-branched-chain amino acid diet producing the highest losses in body fat. The mechanism by which BCAA improves weight reduction alongside caloric restriction is not fully understood, however, it has been theorised that N-enriched hypocaloric diets further increase growth hormone (GH) release, which consequently mobilises fat storage for energy production [89, 90].

### Muscle Damage and Exercise Performance

Sodium citrate supplementation had no effect on exercise performance, however, rapid body mass loss resulted in a significant decrease in mean power. Considering that rapid body mass loss in the range of 5–8% may be accompanied by a significant (36–54%) decrease in muscle glycogen concentration, a reduction in muscle glycogen could have contributed to the decline in performance [91, 92]. While induced metabolic alkalosis would, in theory, be very effective with respect to performance during activities causing extensive perturbations in acid–base balance by reducing the rate of developing muscular fatigue by delaying the decrease in intracellular pH and enhancing muscle energy supply through glycolysis [93], the metabolic alkalosis induced through sodium citrate ingestion did not improve UBISP in the experimental group, which may suggest that the degree of alkalosis was insufficient for enhancing glycolytic ATP production. This could also mean that the disturbance in acid–base balance is not the cause for the decreased performance in UBISP; it may appear that, if sufficient carbohydrate consumption is maintained during rapid body mass loss, blood buffering capacity is not a factor in anaerobic performance. Another point to consider is that the buffering capacity of sodium citrate might be sex-dependent like with sodium bicarbonate, showing greater benefits in men [94]. A few theories have been proposed to explain this: 1) type II muscle fibers mainly rely on glycolysis and females have smaller type II fibers than men [95, 96]; 2) males have a greater glycolytic capacity [97, 98]; and 3) the pH drops to a lesser extent in females than in males during the same type of exercise [97]. Sodium citrate supplementation is also able to induce a significant PV increase. An acute increase in intravascular volume after consuming alkalizing substances has been shown to improve performance in sprinters through better muscle perfusion [99]. While this was not the case in this study, the possibility that exercise performance in athletes could be improved if a sufficient degree of alkalosis is achieved along with an increase in PV makes further research worthwhile.

CrPic supplementation did not improve exercise performance in wrestlers. It was believed that improvements in the cellular uptake and storage of glucose could have a positive effect on metabolic performance through glycogen utilisation [100]. Nonetheless, previous studies have also not reported changes in aerobic or anaerobic performance as a result of enhanced insulin activity and no correlation was able to be made between the increased BM with strength [69, 72]. BCAA supplementation had no effect on CK, CKMB, or LDH levels. These results are inconsistent with previous studies where CK and LDH levels decreased after a dose of 10 gr [101–103]. These inconsistencies might be explained by the type of population studied which were long-distance runners versus trained wrestlers. Other factors that have been shown to affect these enzyme levels include oestrogen, which has a protective effect on the muscle cell membrane that might diminish their increase in blood serum [104]. Citrulline malate supplementation had no effects neither on grip strength nor on strength or agility tests. Citrulline malate supplementation improves ATP production by enhancing ammonia, arginine, and lactic acid buffering mechanisms, which might be useful in reducing fatigue and increasing endurance; however, a longer supplementation period might be needed to produce the desired effects [105, 106].

The study evaluating the effects of HMB-FA supplementation reported significantly lower LDH and CK levels, as well as an increased index of perceived recovery status after the tests; however, the latter two findings are only mentioned, and no data is shown to support these claims. While HMB-FA has been shown to increase intramuscular anabolic signalling, stimulate muscle protein synthesis, and attenuate muscle protein breakdown in humans [107], the effects on markers of muscle damage and perceived recovery following resistance exercise have yielded mixed results in the past [108–111]. Last but not least, the article raises high bias concerns regarding the poor attention to detail in which the article was described, as well as when presenting its results, as previously detailed in Sect. 3.4.

Three studies reported similar effects of CM supplementation on exercise performance. The first one reported enhanced short-term high-intensity exercise performance. Phosphocreatine (PCr) concentrations are higher in fast-twitch muscle fibres compared to slow-twitch muscle fibres, which explains why creatine uptake is helpful in these types of exercises [112]. This effect could also be explained by: 1) an increased intramuscular PCr storage, which increases the amount of exercise to be done before it is depleted and decreases the amount of anaerobic glycolysis required; 2) the suppression of pH reduction in skeletal muscle cells by decreasing the amount of ATP resynthesis made through anaerobic glycolysis [113, 114]. The second study reported similar results, where the muscle endurance test was designed to simulate wrestling conditions by implementing no recovery periods between maximal contractions, but a lower level of intensity between each one. As such, the rate of restoration of physical performance capacity was the intended evaluation. Even though all subjects improved their W_tot_ and W_max_ results in the experimental group compared to the two in the control group, this study only involved 5 subjects in total, which increases the bias of finding significant differences between groups. The third one reported a significant increase in CPK levels, muscular endurance, power, and agility. These results suggest once again that CM supplementation increases intramuscular creatine storage and the ability to reconstitute ATP, which helps create energy reserves that contribute to improved physical performance [115].

Although the two studies evaluating beetroot juice supplementation, acute dose of 140 ml before the test, showed improved balance performance at rest and at fatigue, as well as increased average strength in knee and shoulder exercises, it is worth noting that both studies were written by the same author. While it is true that fatigue has a negative effect on proprioception [116, 117], it can cause poor motor coordination by decreasing muscle stimulation and a gradual decrease in muscle force [118], that nitrate-rich beetroot juice may reduce muscle metabolic perturbation by expanding the antioxidant pool, delay the depletion of ATP reserves, and facilitate muscle glucose and creatine uptake [119–121], there are a couple of limitations with these studies: 1) the first study only induced fatigue in the thigh muscles, but not on other muscles such as hip or leg muscles that also contribute to balance, and 2) both had a small population size.

Iron supplementation, 10 mg/day for 7 days, helped prevent a reduction in endurance capacity during the recovery phase of the rapid weight loss control program. Previous studies have reported that iron deficiency is related to inflammatory and oxidative processes [122, 123], and it also promotes an increased rate of lactate production in muscle [124, 125], which could be prevented with iron supplementation.

Arginine supplementation, ranging from 1.2 to 2 g/kg with different durations, reduces the O_2_ cost of moderate-intensity cycle exercise, and the VO_2_ slow component amplitude, and increases the time to task failure in severe intensity exercise. L-arginine is a main element for the synthesis of NO, NO synthase converts L-arginine into NO and L-citrulline in the presence of some cofactor [126]. NO is a potent vasodilator which acts by increasing cyclic guanosine monophosphate (cGMP), which in turn causes the relaxation of smooth muscle that consequently increases perfusion causing better performance and post-exercise recovery [126, 127]. While caffeine supplementation was able to reduce the PWPT time in this article, other articles show contradictory evidence for supplementation with caffeine, with not only neutral but also negative effects. On the one hand, caffeine has an ergogenic effect, in which there is a calcium-induced calcium release by acting on the ryanodine receptor that enhances calcium signalling, as well as the extracellular secretion of proteins such as myokines [128]. On the other hand, the ergogenic effects of caffeine can be variable between individuals because of a polymorphism in the CYP1A2 gene that codifies a protein that metabolises caffeine into paraxanthine and methylxanthines, which might prove detrimental for some individuals [129, 130].

### Hormonal and Metabolic Markers

Even though the addition of sodium citrate to a high carbohydrate diet increased blood pH, blood buffering capacity, and PV during the 16 h recovery period after rapid body mass loss, it did not affect exercise performance. CrPic supplementation did not have any effect on fasting serum blood glucose and insulin concentrations. It was believed that improvements in the cellular uptake and storage of glucose through insulin potentiation or increased insulin sensitivity could improve glycogen mobilisation and breakdown, as well as reduce the concentrations of fasting insulin and glucose in the blood [100]. This absence of change might be explained because of the heightened insulin sensitivity in highly trained groups of people such as professional wrestlers [131]. With CM supplementation, plasma ammonia concentrations did not significantly differ between groups, although they did decrease significantly after exercise, a known effect after short-term high-intensity exercise [132]. Lactate and glucose levels did not vary between groups, which suggests that the mobilisation of carbohydrates is not dependent on this supplement during the recovery period of rapid body mass loss. There was no effect of the exercise test on plasma urea concentrations, even though it increased significantly after rapid body mass loss, which reflects an increased rate of protein degradation [133]. On the other hand, the whole-body creatine retention levels were not found to be as expected in the experimental group. It has been reported that a single 5 g dose of CM considerably raises the concentration of creatine in plasma for at least 2 h [134]. Therefore, it can be assumed that the short consumption period of the last dose (around two hours before performing the last test) probably resulted in the last dose not reaching the muscle.

Supplementation with a single dose of arginine increased total amino acid (TAA) concentrations following exercise in both trials, which could be explained by haemoconcentration, however, pre- and post-exercise TAA concentrations were still significantly higher in the supplementation group. These changes could be helpful with fatigue. The causes of fatigue are complex, and influenced by events occurring in both the periphery and the central nervous system [135]. A theory of fatigue developing from the central nervous system is based on the observation that exercise promotes an increase in plasma-free fatty acids and a decrease in large neutral amino acids such as leucine, methionine, valine, phenylalanine and tyrosine due to uptake by skeletal muscle [136]. As both conditions favour more tryptophan entering the CNS, increased production of brain serotonin might be expected, and this could account for the decreased motor drive and increased sensation of fatigue [137]. A significant increase in plasma concentrations of all these large neutral amino acids was observed, except for valine (probably because of the small study population), which might delay the effects of central fatigue. It has also been reported that the plasma BCAA increase produced by arginine administration may attenuate fatigue because BCAAs and free tryptophan compete for transport through the blood–brain barrier [138]. Citrulline supplementation was not able to reduce hypoxanthine or HGPRT levels. Hypoxanthine is derived from the degradation of purines and can indicate metabolic stress in the muscles; while it is not frequently used, it can directly correlate with the amount of ATP consumed inside the cell, making it a theoretically good marker of muscle fatigue [139]. On the other hand, HGPRT levels are also not a commonly used biomarker for muscle damage, but have been used in muscle dystrophies research [140]. Similar to the results obtained in exercise performance, a high but acute dose might not be sufficient to bring down their levels, which opens the possibility of different doses and longer durations of supplementation.

Spirulina supplementation caused a significant decrease in MST concentrations that can mediate FM reduction. MST inhibition results in adipose tissue loss in high-fat diet-induced mice [141, 142]. Also, the catabolic state decreases with lower levels of FST and increases with higher MST concentrations, resulting in a diminished FST:MST ratio (which is present in the study) [143]. The lower levels of liver enzymes might be caused by the presence of beta carotene, superoxide dismutase, and phycocyanin, all of which reduce cell damage, induce the regeneration of damaged hepatocytes, and also reduce oxidative stress and inflammation [144, 145].

Thyme tea three times a day (with a loading dose of 150 cm^3^ for 35 days), might help improve exercise performance by increasing antioxidant capacity and increasing the resistance to oxidative stress [146, 147]. The study investigating the effects of carbohydrate, BCAA, and arginine supplementation reported significantly higher concentrations of glucose and insulin due to the impaired insulin-dependent glucose disposal and glycogen synthesis in skeletal muscle caused by the increase in the inhibitory insulin receptor substrate-1 phosphorylation and decreasing PI3K activity [148]. Iron supplementation significantly decreased lactate levels, suggesting that it increased the efficacy of muscle contractions by improving the maximum oxygen uptake [56]. Supplementation might prevent iron deficiency, which causes the iron-containing enzymes in skeletal muscle and liver to be altered and promote an increased rate of lactate production [124, 125].

### Other Outcomes

The study that investigated thyme tea supplementation reported a significant decrease in malondialdehyde (MDA) levels. MDA is one of the indicators of oxidative stress and one of the main products of lipid peroxidation [147]. As such, thyme tea might be able to reduce oxidative stress in the muscles and improve exercise performance. While IHE significantly increased NO, H_2_O_2_, tissue regeneration mediators, and multiple haematological parameters, it appears as if hypoxic exposure is the key factor for these changes. When compared to the other groups, arginine alone was not sufficient to elicit significant results in any of the measured outcomes. Nonetheless, the combination of IHE and arginine supplementation was superior to IHE alone in most cases [40]. Whey protein supplementation resulted in significantly higher total protein and albumin levels. Whey protein has one of the highest amounts of BCAA, which promotes the signalling pathways of muscle protein synthesis and also serves as a donor of nitrogen to alanine and glutamine during protein modulation [149].

### Limitations and Future Perspectives

One limitation of this review was the heterogeneity between studies since some involved either multiple sports or underage participants. In addition, the type of supplementation, but also in dose, frequency, or duration of administration, as well as in the measured outcomes, considerably differed. It is also worth mentioning that some authors appear on multiple studies, for example, Tlatici, A. was the main author for both BRJ articles [51, 58]; Ööpik, V. and Timpman, S. were involved in two CM articles as well as in the sodium citrate article [37, 50]; Jun-Young Sung was the main author for both Spatone® and iron supplementation articles [55, 56]; and Ulas, H. was the first author for two arginine articles [52, 53].

As such, future research in this area can be improved: 1) studies should ensure they are sufficiently powered to detect statistically significant group effects by performing a priori sample size calculations; 2) more studies focusing on wrestling are needed. Each type of sport demands specific abilities, which makes the measurement of exercise performance outcomes uneven since the results are usually mixed between participants; 3) underage participants should not be included in the same studies as adults since the metabolism and excretion of certain substances, as well as their performance outcomes, are more likely to differ, providing confusing results.

## Conclusions

Based on the findings from the current systematic review, there is some evidence that:

I) Sodium citrate supplementation has a positive effect on BM re-gain [37].
II) Supplementation with CrPic has no significant effects in BC, MDEP, HMM or OO [38].
III) CM supplementation generally improved exercise performance but had mixed outcomes regarding weight gain [29, 39, 43, 50].
IV) Arginine supplementation significantly increased NO and HGF levels, but had mixed outcomes regarding exercise performance [40, 52–54].
V) BCAA supplementation showed mixed results regarding glucose and insulin levels, but had significant effects on reducing body and fat mass; lastly, it generally had no significant effect on exercise performance [45, 57].
VI) Beetroot juice generally improves exercise performance [51, 58].
VII) HMB-FA supplementation requires further investigation since the results of the included article possess a high risk of bias [41].
VIII) Spirulina supplementation helped decrease BM, BFP, FM and SMM [42].
IX) Supplementation with iron had contradictory outcomes: one study reported that VO_2_max increased while the other reported it decreased; interestingly, similar to sodium bicarbonate supplementation, it appears iron supplementation might be sex dependent [55, 56].
X) Thyme tea supplementation exhibited a meaningful increase in TAC and lower MDA levels [46].
XI) Green and oolong tea extracts were useful for body mass loss [47].
XII) Whey protein supplementation had generally no significant effect on exercise performance [48].
XIII) Supplementation with caffeine enhanced exercise performance [49].

Overall, this review might be useful for the creation of safer, evidence-based weight-cutting protocols compared to the standard practices used today, however, more studies are needed to carefully determine whether each type of supplementation is helpful for modifying body composition, physiological status, or exercise performance in wrestlers.

## Key References

- Jafari RA, Hosseini S, Rashidlamir A, Nobari H. Evaluating the Impact of Active and Passive Recovery Strategies and Citrulline-Malate Supplementation in Wrestling: Do the Results Add Up? Acta kinesiologica. 2024 08/09;18.
  - This study examined the effects of active versues passive recovery and citrulline malate supplementation on performance and biomarkers in trained wrestlers during a simulated tournament. While no overall significant effects were found, differences in HGPRT levels, agility, and perceived exertion suggest that recovery strategies may influence specific aspects of performance.
- Bagheri R, Negaresh R, Motevalli MS, Wong A, Ashtary-Larky D, Kargarfard M, et al. Spirulina supplementation during gradual weight loss in competitive wrestlers. Br J Nutr. 2022 Jan 28;127(2):248–56.
  - This study highlights that spirulina supplementation during gradual weight loss enhances fat loss, reduces myostatin and liver enzyme levels, and helps maintain IGF-1 and follistatin concentrations in competitive wrestlers. These findings suggest that spirulina may be beneficial for optimizing body composition and metabolic markers during weight reduction.
- Zahabi G, García Ramos A, Ilic V, Nedeljkovic A, Štajer V, Žugaj N, et al. Effects of Short-Term Creatine Monohydrate Supplementation Combined with Strength Training on the Physical Fitness Characteristics and Muscle Hypertrophy in Junior Women Wrestlers. Journal of Health and Allied Sciences NU. 2024 07/29.
  - This study demonstrates that short-term creatine supplementation, combined with strength training, significantly enhances muscle hypertrophy and physical fitness in junior female wrestlers. The findings suggest that creatine could be a valuable addition to strength training programs for improving athletic performance in this population.

## Data Availability

No datasets were generated or analysed during the current study.

## Abbreviations

AA: Amino acid
AE: Average extension
AER: Average external rotation;
AF: Average flexion
AIR: Average internal rotation
ALT: Alanine aminotransferase
AP: Average power
APSI: Anterior-posterior stability index
AST: Asparte aminotransferase
AT: Adipose Tissue
BAT: Burpee agility test
BC: Body composition
BCAA: Branched-chain amino acids
BDNF: Brain derived neurotrophic factor
BE: Base excess
BF: Body fat
BFP: Body fat percentage
BLC: Back-leg-chest
BM: Body mass
BMI: Body mass index
BRJ: Beetroot juice
BW: Body weight
CH: Carbohydrate
CK: Creatine kinase
CKMB: Creatine kinase- MB
CM: Creatine monohydrate
CPK: Creatine phosphokinase
CRP: C-reactive protein
EPO: Eritropoyetin
Fe: Iron
FFM: Fat free mass
FI: Fatigue index
FlexAvg: Average flexion
FlexP: Peak flexion
FM: Fat mass
FST: Follistatin
GLC: Glucose:
GTE: Green tea extract
H2O2: Hydrogen peroxide
Hb: Haemoglobin
hBCAA: Hypocaloric BCAA group
HCO_3_: Bicarbonate
HDL: High density lipoprotein
HGF: Hepatocyte growth factor
HGPRT: Hypoxanthine-guanine phosphoribosyltransferase test
HMB-FA: β-Hydroxy-β-methylbutyrate
HMM: Hormonal and metabolic markers
HR: Heart rate
HTC: Haematocrit
IAT: Illinois agility test
IGF-1: Insulin-like growth factor 1
IL: Interleukin
JSWPT: Jafari's simulated wrestling performance test
LBM: Lean body mass
LDH: Lactate deshydrogenase
LDL: Low density lipoprotein
MEB: Mean energy balance
MCH: Mean corpuscular haemoglobin
MCHC: Mean corpuscular haemoglobin concentration
MCV: Mean corpuscular volume
MDA: Malondialdehyde
MDEP: Muscle damage and exercise performance
MLSI: Medial-lateral stability index
MP: Mean power
MST: Myostatin
MT: Muscle tissue
MVC: Maximal isometric volume contraction
N/S: Not specified
NEFA: Non-esterified fatty acid
NO: Nitric oxide
OO: Other outcomes
OSI: Overall stability index
OTE: Oolong tea extract
PDGF: Platelet derived growth factor
Peak AnP: Peak anaerobic power
PE: Peak extension
PER: Peak external rotation
PF: Peak flexion
PIR: Peak internal rotation
PP: Peak power
PRS: Perceived recovery status
PWPT: Pittsburgh wrestling performance test
RBC: Red blood cells
RDW: Red cell distribution width
REL Anc: Relative anaerobic capacity
REL VOmax: Relative peak aerobic power
RET: Reticulocytes
RHR: Reserve heart rate
RM: Repetition maximum
RPE: Rating of perceived exertion
RPF: Rating of perceived fatigue
RSH: Total sulfhydryl group
SAT: Subcutaneous adipose tissue
SMM: Skeletal muscle mass
T3: Triiodothyronine
TAA: Total aminoacid concentration
TAC: Total antioxidant capacity
TC: Total cholesterol
TG: Triglycerides
TIBC: Total iron binding capacity
TNF-alpha: Tumor necrosis factor alpha
UBISP: Upper body intermittent sprint performance test
USG: Urine specific gravity
VAT: Visceral adipose tissue
VEGF: Vascular endothelial growth factor
VJT: Vertical jump test
WCV: White cell volume
Wmax: Maximum work
Wtot: Total work
